# Feasibility of remote neurocognitive assessment: pandemic adaptations for a clinical trial, the Cognition and Obstructive Sleep Apnea in Parkinson’s Disease, Effect of Positive Airway Pressure Therapy (COPE-PAP) study

**DOI:** 10.1186/s13063-021-05879-1

**Published:** 2021-12-11

**Authors:** Annie C. Lajoie, Joelle Crane, Ann R. Robinson, Anne-Louise Lafontaine, Andrea Benedetti, R. John Kimoff, Marta Kaminska

**Affiliations:** 1 Respiratory Epidemiology and Clinical Research Unit, Research Institute of the McGill University Health Centre, Montreal, QC Canada; 2 Montreal Neurological Hospital, McGill University Health Centre, Montreal, Canada; 3 Deparment of Medicine and Dept. of Epidemiology, Biostatistics & Occupational Health, McGill University, Montreal, Canada; 4 Respiratory Division & Sleep Laboratory, McGill University Health Centre, Montreal, QC Canada

**Keywords:** Parkinson’s disease, Sleep disordered breathing, Obstructive sleep apnea, Neurocognitive testing, Cognitive function

## Abstract

**Background:**

The COVID-19 pandemic poses challenges for timely outcome assessment in randomized clinical trials (RCT). Our aim was to describe our remote neurocognitive testing (NCT) protocol administered by telephone in patients with Parkinson’s disease (PD) and obstructive sleep apnea (OSA).

**Methods:**

We studied PD patients with OSA and Montreal Cognitive Assessment (MoCA) score ≤ 27 participating in a RCT assessing OSA treatment impact on cognition. Trial outcomes included change in MoCA and specific cognitive domains from baseline to 3 and 6 months. With COVID19 pandemic-related restrictions, 3-month visits were converted from in-person to telephone administration with materials mailed to participants for compatible tests and retrieved by courier the same day. In exploratory analyses, we compared baseline vs. 3-month results in the control arm, which were not expected to change significantly (test-re-test), using a paired *t*-test and assessed agreement with the intraclass correlation coefficient (ICC).

**Results:**

Seven participants were approached and agreed to remote NCT at 3-month follow-up. Compared to the in-person NCT control arm group, they were younger (60.6 versus 70.6 years) and had a shorter disease course (3.9 versus 9.2 years). Remote NCT data were complete. The mean test-retest difference in MoCA was similar for in-person and remote NCT control-arm groups (between group difference − 0.69; 95%CI − 3.67, 2.29). Agreement was good for MOCA and varied for specific neurocognitive tests.

**Conclusion:**

Telephone administration of the MoCA and a modified neurocognitive battery is feasible in patients with PD and OSA. Further validation will require a larger sample size.

**Supplementary Information:**

The online version contains supplementary material available at 10.1186/s13063-021-05879-1.

## Introduction

Parkinson’s disease (PD) is the fastest growing neurodegenerative disease [1]. Non-motor manifestations, such as cognitive impairment, are increasingly recognized as major detrimental factors of health-related quality of life in PD [2]. It is estimated that PD-related dementia will affect up to 80% of PD patients, causing substantial societal and health care burden and increased mortality [3–5]. Factors external to PD, such as obstructive sleep apnea (OSA), may further impact the evolution of cognitive dysfunction in this population. OSA, characterized by recurrent complete (apnea) or partial (hypopnea) upper airway obstruction during sleep, is a frequent comorbidity in PD, where it affects 20–60% of patients [6]. OSA has been proposed as a potential risk factor for cognitive decline and for dementia in the general population [7]. In PD, OSA has been associated with worse cognitive function, and observational studies looking at treatment of OSA suggest cognition may improve with therapy [6, 8].

The Montreal Cognitive Assessment (MoCA) test is widely used to evaluate global cognition and to screen for cognitive impairment in the general population, as well as in patients with neurodegenerative diseases [9–11]. In PD, the MoCA has the best diagnostic accuracy to detect mild cognitive impairment and dementia, compared with other screening tools such as the Mini-Mental State Examination (MMSE) [10]. Consequently, global cognitive function in PD is usually monitored during in-person medical visits using the MoCA test [12, 13]. However, Telemedicine has gained in popularity with the evolution of technology and even more so due to the recent SARS-CoV-2 and related disease (COVID-19) pandemic [14]. Furthermore, even outside of a pandemic, Telemedicine has the potential to alleviate the additional burden imposed by research activities on patients with PD, who have to engage in frequent medical visits with numerous healthcare providers (e.g., neurologist, occupational therapy) as part of their regular follow-up. However, there are additional barriers to the utilization of web-based videoconference assessments in PD patients, who often have motor, visual, and cognitive impairment. Telephone administration of the MoCA mays thus be a more convenient way to remotely test for cognitive dysfunction in PD. Telephone administration of a modified MoCA was found to reliably identify mild cognitive impairment following stroke or transient ischemic attacks but has never been studied in PD [15–17].

We aimed to describe and evaluate the feasibility of our remote cognitive testing protocol implemented during the first wave of the COVID-19 pandemic (as of March 2020) in a cohort of PD patients with OSA and reduced cognitive function who were randomized to the control arm of a RCT looking at the effect of OSA treatment on cognitive function in PD. The exploratory aim was to assess the agreement between the remote and in-person MoCA testing, and on tests of specific domains of neurocognitive function.

## Methods

### Participants

The COPE-PAP study (NCT02209363) is a randomized controlled trial conducted in Montreal (Quebec, Canada), where the primary outcomes is change in global cognition (Montreal Cognitive Assessment [MoCA]) following 6 months of treatment (positive airway pressure therapy or PAP) versus control nasal dilator strips (NDS) in PD patients with OSA and reduced cognition (MOCA score ≤ 27). OSA is defined as a respiratory disturbance index ≥15 events per hour on polysomnography (PSG). The usual protocol involves three neurocognitive assessments—at baseline, 3-month, and 6-month follow-up. In addition to the MoCA, and the neurocognitive testing (NCT) battery includes tests of attention and working memory, executive functions, language, visuospatial abilities, and memory. A different version of the MoCA is administered at each visit (version 1.0 at baseline and version 2.0 at 3 months) to reduce learning effects [18, 19].

Treatment with PAP has been found to improve or prevent the decline of cognitive function in patients with OSA from the general population and in those with neurodegenerative or neurological disorders [20–22]. For this reason, only individuals from the COPE-PAP trial randomized to the control group were included in the test-retest analysis, as we do not expect their cognitive function to improve. While the choice of comparator is debated, there is no “true” placebo alternative to CPAP. Most studies employ either nasal dilator strips or sham-positive airway pressure (sham-PAP). However, sham-PAP delivers a certain (low) amount of pressure which may have physiologic effects on OSA and may adversely impact sleep architecture [23]. Most studies evaluating nasal dilator strips suggest they do not alter OSA or sleep parameters (e.g., apnea-hypopnea index or oxygen desaturations) [24]. For this reason, nasal dilator strips were chosen as the comparator in the COPE-PAP trial.

### Remote neurocognitive testing procedure

Because of COVID-19-related sanitary measures, in-person research visits were suspended. To preserve time-sensitive study data collection, a decision was made to conduct the 3-month assessment remotely via telephone evaluation. Baseline and 6-month visits, instrumental for the assessment of the study’s primary outcome, were delayed but conducted in person. A procedure for remote NCT was approved by our institution’s research ethics board. Participants scheduled to have their 3-month NCT at that time were contacted to assess willingness to participate in remote NCT or forgo the evaluation. All seven concerned participants (3 in the treatment group and 4 in the control group) agreed to telephone NCT.

A few days before testing, the research coordinator provided the participants with verbal and written (email) instructions on the procedures pertaining to the telephone neurocognitive assessment. The participants received a sealed double envelope containing the required testing materials and questionnaires via courier a few days ahead of testing. All testing materials were quarantined for a minimum of 48 h before the testing session. Participants were instructed to discard the outer envelope, engage in hand hygiene, and then wait for the neuropsychologist’s (tester) instructions before opening the testing material. The latter was sealed in an inner envelope to prevent participants from rehearsing or learning the tests.

The neuropsychologist contacted the participant before the testing session to establish the clarity of the telephone connection, to review instructions, and to confirm the planned date and time of testing. Assessments were scheduled in order to preserve the usual time of testing for each patient (morning versus afternoon). The participants would then complete the remote cognitive assessment and questionnaires following the tester’s instructions delivered over the phone. At the end of the testing session, participants were instructed to seal all the testing material in the provided pre-addressed return envelope. The testing material was sent back to the study site via courier immediately after completion. Participants were also mandated not to copy the testing material and to perform the evaluation without help from their caregiver.

### Adapted neurocognitive testing

Global cognitive function was assessed using the MoCA and conducted in accordance with current recommendations (mocatest.org) with the exception that the visuospatial/executive and naming (visual component) test materials were mailed. Participants could record their answers directly on the form for the visuospatial component and see the naming stimuli, for which they provided answers orally [12].

Specific tests for domains of cognitive function included the Rey Auditory Verbal Learning Test (RAVLT), the Digit Span test (WMS-III), the Symbol Digit Modalities Test (SDMT), and the Delis-Kaplan Executive Function System (D-KEFS) subtests for verbal fluency and color-word interference. Permission for distribution of original study material for the SDMT and D-KEFS color-word interference for testing according to this protocol was obtained from both test distributors [WPS (Torrance CA, USA) and Pearson Clinical Assessment (Toronto, Canada) respectively]. The RAVLT, Digit Span test, and D-KEFS verbal fluency subtest were administered remotely following usual procedure, with the participants’ oral answers directly recorded by the neuropsychologist on the tester’s form. However, because visual stimuli are required for the SDMT and the D-KEFS color-word interference subtest, original forms were sent to the participants, allowing them to respond orally according to the tester’s instructions, and their answers were recorded by the neuropsychologist on the test form. Test materials were only exposed to the participant for the duration of the test, as participants were instructed to turn the page face down after completion. Patients also completed questionnaires on subjective daytime sleepiness (Epworth Sleepiness Scale (ESS)), quality of life (Parkinson Disease Quality of Life (PDQ39)), quality of sleep (Parkinson Disease Sleep Scale (PDSS)), depressive symptoms (Beck Depression inventory II (BDI-II)), and questionnaire items from the Movement Disorder Society Unified Parkinson’s Disease Rating Scale (MDS-UPDRS), asking for clarification as needed. The RAVLT delayed recall and recognition were administered after the questionnaires to maintain a similar delay period to that of in-person NCT. Tests that could not be done remotely included the D-KEFS trail making test, Hooper visual organization test, Boston naming test, and psychomotor tapping test.

Feasibility was assessed in all participants undergoing remote NCT (treatment and control group) by evaluating participants’ acceptance of the procedure and evaluating any drawbacks with respect to data acquisition.

### Statistical analysis

Participants were divided into two groups based on their follow-up NCT testing: remote or in-person. Baseline and 3-month scores in MoCA and other NCT scores were compared using a paired *t*-test. The between-group difference was assessed using an unpaired *t*-test with Welch’s correction. Data are reported as mean and standard error of the mean (SEM). The agreement between baseline and 3-month scores in MoCA (total score and score on visual and verbal components) was estimated using the intraclass correlation coefficient (ICC) and its associated 95% confidence interval (CI). Threshold for reliability was defined as follows: excellent agreement (ICC ≥ 0.75), fair to good agreement (0.40 < ICC < 0.75), and poor agreement (ICC ≤ 0.40). Agreement was similarly assessed for other NCT.

## Results

### Participant characteristics

Four participants (3 men and 1 woman) from the COPE-PAP trial randomized to the control group and 3 participants randomized to the treatment group (2 men and 1 woman) underwent 3-month NCT remotely because of COVID sanitary measures. Twenty-five participants (16 men and 9 women) randomized to the control group had both baseline and follow-up NCT assessments in-person. Participants in the remote NCT cohort were younger and had a shorter disease course and more years of education compared to the in-person NCT group (Table 1). The levodopa equivalent daily dose and the MDS-UPDRS total score as well as the non-motor experience of daily living component were lower in the remote NCT, compared to the in-person NCT cohort. The motor component MDS-UPDRS scores were similar between groups, as were the baseline average MoCA, Epworth Sleepiness Scale scores, and OSA severity.

**Table 1:** Baseline characteristics of participants

Characteristics	Remote NCT cohort (control or CPAP) (*n* = 7)	COPE-PAP control cohort (*n* = 25)
Age, years	60.6 (4.8)	70.6 (10.1)
Sex (M/F)	5/2	16/9
BMI, kg/m^2^	30.4 (6.8)	29.7 (4.3)
Education, years	16.3 (2.6)	14.4 (3.2)
MoCA test score	23.9 (1.8)	22.4 (3.0)
Epworth Sleepiness Scale score	10.9 (3.0)	10.6 (4.8)
Time from PD diagnosis, years	3.9 (2.2)	9.2 (7.0)
Levodopa equivalent dose, mg/day	374.9 (152.0)	781.9 (446.8)
MDS-UPDRS total score	52.0 (12.2)	64.9 (17.1)
MDS-UPDRS part 1 (non-motor experience of daily living)	12.4 (5.9)	14.2 (4.7)
MDS-UPDRS part 3 (motor examination)	29.1 (7.8)	31.2 (9.8)
Sleep apnea variables		
AHI, events/hour	39.8 (11.2)	40.4 (29.7)
ODI, events/hour	14.1 (9.6)	16.3 (18.8)
Mean SpO_2_	94.3 (1.6)	94.1 (1.9)
Nadir SpO_2_	86.7 (5.1)	86.2 (7.7)
T90, % TST	1.0 (1.4)	2.5 (4.3)

Results are in mean (standard deviation). *AHI*, Apnea-hypopnea index, *BMI*, Body mass index, *MDS-UPDRS*, Movement Disorder Society- Unified Parkinson’s Disease Rating Scale, *MoCA*, Montreal Cognitive Assessment, *NCT*, Neurocognitive testing; *ODI*, Oxygen desaturation index, *RDI*, Respiratory disturbance index, *T90*, % time spent with SpO_2_ < 90%; *TST*, Total sleep time

### Feasibility

All the participants scheduled to have their 3-month NCT during the COVID-19 lockdown agreed to be tested remotely over the phone. There were no difficulties with delivery of test materials to or from participants, nor with scheduling of test calls in the remote NCT group. For the tests that could be performed remotely and reported here, there were no missing data in either the remote or in-person NCT group. In the remote NCT cohort, one participant received help from his caregiver during the verbal fluency test. This answer, overheard by the neuropsychologist, was subtracted, and instructions were reiterated to the participant. No other limitations occurred during remote telephone testing.

### Agreement between remote and in-person MoCA

**Fig. 1:**
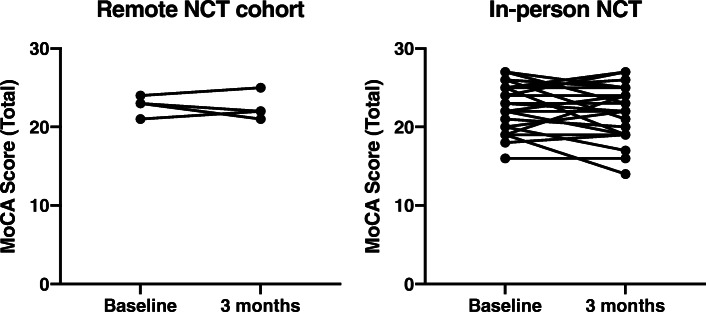
Change in total MoCA score over time. Individual change from baseline to 3 months in total MoCA score in the remote NCT, control arm cohort (*n* = 4, left panel) and in-person control arm cohort (*n* = 25, right panel). In the remote NCT group, the total MoCA score was 22.75 ± SD: 1.26 at baseline and 23.00 ± 2.71 at 3 months (mean change 0.25, SEM 1.11). In the in-person NCT group, the MoCA score was 22.40 ± 3.00 and 21.96 ± 3.40 at baseline and 3 months respectively (mean change −0.44, SEM 0.55). The mean difference in change from baseline between groups was −0.69 (95% confidence interval − 3.67 to 2.29). MoCA, Montreal Cognitive Assessment Test; NCT, neurocognitive testing

The mean difference between baseline and 3 months total MoCA scores was 0.25 (SEM 1.11) in the remote NCT group and − 0.44 (0.55) in the in-person NCT group (Fig. 1 and Table 2). The mean difference in change between groups was − 0.69 [95% CI − 3.67 to 2.29]. The performance on the visual and verbal components of the MoCA was also similar between timepoint within groups and between groups.

**Table 2:** Change in MoCA and neurocognitive domains (test-retest) in the remote and in-person NCT cohorts in control arm participants

Component (maximum score possible)	Remote NCT cohort (*n* = 4)			In-person NCT cohort (*n* = 25)			Between groups	
	Baseline (in-person)	3 months (remote)	Mean change (SEM)	Baseline (in-person)	3 months (in person)	Mean change (SEM)	Mean difference in change (SEM)	95% CI
Total MoCA score (30)	22.75 (1.26)	23.00 (2.71)	0.25 (1.11)	22.40 (3.00)	21.96 (3.40)	− 0.44 (0.55)	− 0.69 (1.45)	− 3.67;2.29
Visual component (8)	6.25 (0.96)	6.00 (0.82)	− 0.25 (0.25)	6.04 (1.34)	6.04 (1.40)	0.00 (0.23)	0.25 (0.59)	− 0.96; 1.46
Verbal component (22)	16.25 (0.50)	16.25 (1.50)	0.00 (0.58)	16.16 (2.46)	15.72 (2.53)	− 0.44 (0.53)	− 0.44 (1.36)	− 3.23; 2.35
Neurocognitive domains testing								
Attention and working memory								
Digit span—longest forward	0.04 (1.69)	0.22 (1.73)	0.19 (0.45)	− 0.41 (0.70)	− 0.15 (0.93)	0.26 (0.13)	0.07 (0.37)	− 0.68; 0.82
Digit span—longest backward	0.26 (0.42)	− 0.21 (0.94)	− 0.47 (0.53)	− 0.26 (0.67)	− 0.25 (0.71)	0.01 (0.13)	0.48 (0.38)	− 0.30; 1.26
Digit span total (forward and backward)	0.28 (1.14)	0.28 (1.17)	0.00 (0.24)	− 0.28 (0.76)	− 0.24 (0.79)	0.04 (0.09)	0.04 (0.24)	− 0.46; 0.54
SDMT	− 1.24 (1.46)	− 0.88 (1.46)	0.36 (0.15)	− 1.42 (1.06)	− 1.19 (1.23)	0.23 (0.19)	− 0.13 (0.49)	− 1.13; 0.87
Total immediate recall (RAVLT)	− 0.78 (0.78)	0.43 (1.42)	1.21 (0.69)	− 1.00 (0.98)	− 0.87 (0.99)	0.13 (0.19)	− 1.08 (0.54)	− 2.19; 0.03
Executive functions								
Category switching accuracy (verbal fluency)	− 0.50 (1.39)	− 0.77 (2.00)	− 0.27 (0.55)	− 0.38 (1.18)	− 0.35 (1.33)	0.03 (0.24)	0.30 (0.64)	− 1.01; 1.61
CWI inhibition	0.62 (0.82)	0.62 (0.72)	0.00 (0.26)	− 0.15 (1.20)	− 0.03 (1.11)	0.12 (0.14)	0.12 (0.37)	− 0.63; 0.87
CWI inhibition/switching	− 1.19 (1.61)	− 0.33 (1.99)	0.86 (0.63)	− 0.87 (1.54)	− 0.76 (1.69)	0.11 (0.18)	− 0.75 (0.51)	− 1.80; 0.30
Language								
Verbal fluency—letter	− 0.79 (0.84)	− 0.62 (0.87)	0.18 (0.23)	− 0.46 (0.92)	− 0.36 (1.09)	0.11 (0.17)	− 0.07 (0.44)	− 0.97; 0.83
Verbal fluency—category	− 0.70 (1.01)	− 0.34 (1.70)	0.35 (0.36)	− 0.52 (1.10)	− 0.69 (1.17)	− 0.17 (0.14)	− 0.52 (0.38)	− 1.30; 0.26
CWI—color naming	− 0.77 (1.30)	− 0.70 (1.24)	0.07 (0.37)	− 0.60 (1.36)	− 0.37 (1.19)	0.23 (0.11)	0.16 (0.31)	− 0.48; 0.80
CWI—word reading	− 0.17 (0.74)	− 0.18 (0.47)	− 0.01 (0.14)	− 0.31 (1.31)	− 0.19 (1.25)	0.12 (0.06)	0.13 (0.16)	− 0.20; 0.46
Memory								
Delayed recall (RAVLT)	− 1.28 (0.73)	0.12 (1.40)	1.40 (0.52)	− 1.08 (1.02)	− 1.10 (0.99)	− 0.03 (0.15)	− 1.43 (0.42)	− 2.30; − 0.56*

MoCA test scores are presented as mean (standard deviation or SD) and neurocognitive domains scores as mean Z-scores (SD). Within-group paired *t*-tests were not statistically significant (*p* > 0.05). *CI* Confidence interval, *CWI* Color-Word Inhibition, *MoCA* Montreal Cognitive Assessment, *NCT*, Neurocognitive testing, *RAVLT* Rey Auditory Verbal Learning Test, *SDMT* Symbol Digit Modality Test, *SEM* Standard error of the mean

The estimated agreement between the in-person and remote MoCA total score was fair to good in the remote NCT cohort (ICC 0.60; 95% CI − 0.43 to 0.97) and the in-person NCT cohort (ICC 0.64; 95% CI 0.34 to 0.82) (Supplemental Table 1). There was an excellent agreement between the baseline and 3-month visual component scores of the MoCA (ICC 0.84, 95%CI 0.08 to 0.99) in the remote NCT group, and fair to good agreement in the in-person NCT cohort (ICC 0.65, 95% CI 0.36 to 0.83). The verbal component of the MoCA for both groups displayed fair to good agreement between testing sessions (Supplemental Table 1).

### Agreement between remote and in-person assessment of neurocognitive domains

In most of the domains, the performance (*Z* score) remained stable between baseline and 3 months in both groups, without significant difference between groups (Table 2). However, the performance on the delayed recall (RAVLT; memory domain) improved substantially in the remote NCT group [(baseline − 1.28 (SEM0.73) and 3 months 0.12 (1.40), mean change 1.40 (0.52)] and remained relatively constant in the in-person NCT group [baseline − 1.08 (1.02) and 3 months − 1.10 (0.99), mean change − 0.03 (0.15)], with a statistically significant mean difference in change between groups [− 1.43 (0.02); 95% CI − 2.30 to − 0.56].

The agreement between the baseline and 3-month NCT was excellent for most tasks in the remote NCT group, with the exceptions of the digit span backwards (attention and working memory domain; ICC − 0.21 [95%CI − 0.66 to 0.72]), total immediate recall (attention and working memory domain; ICC 0.21 [95%CI − 0.66 to 0.87]), and delayed recall on the RAVLT (memory; ICC 0.18 [95%CI − 0.67 to 0.86]) in the remote NCT cohort (Supplemental Table 1). In the in-person NCT cohort, agreement between baseline and 3 months was fair to good or excellent.

## Discussion

The COVID-19 pandemic has led to the emergence of widespread use of Telemedicine in the evaluation and monitoring of many diseases, and the field of movement disorders has not been immune to such needs [14]. The primary goal of this study was to describe and evaluate the feasibility of our methods used for telephone administration of the MoCA and neurocognitive assessment tasks implemented during the COVID-19 pandemic.

To our knowledge, this is the first study evaluating the feasibility of telephone administration of the MoCA in patients with PD, with mailing of test materials to participants. Our results suggest that this method is feasible in the context of a RCT and well-accepted by participants. It could be an alternative to in-person testing in patients with PD and mild cognitive impairment. Remote telephone NCT has advantages and also disadvantages (Table 3). An important advantage is that it eliminates costs as well as time and constraints related to travel to and from the testing site, for participants and potentially caregivers. Furthermore, being tested in a familiar environment may reduce participants’ anxiety. Remote NCT via Telemedicine may allow to increase participation in research studies of patients with mobility or transport issues who might otherwise not have been involved in studies involving in-person visits, which could enhance external validity.

**Table 3:** Advantages and disadvantages of telephone neurocognitive assessment in research

	Patient perspective	Study perspective
Advantages	- Eliminates costs and time related to travel -May reduce caregiver burden related to travel for testing - Provides a familiar testing environment -Reduces anxiety regarding study visit (e.g., being late, finding room)	- May increase willingness to participate in protocol. -May allow inclusion of individuals who would otherwise be unable to attend (e.g., disabled, living far, limited transport options…), broadening the study sample and enhancing external validity of study results. -Mitigates data loss when participant is unable to attend visit physically (e.g., COVID-19 pandemic)
Disadvantages	- Coordination with study personnel requiring multiple phone calls - No visual clues (especially for those with hearing impairment) -No research staff available in person to help with testing materials, potentially anxiogenic -Possible distractions, e.g., other household members, noise	- Time-consuming Logistics (scheduling phone call, sending material to and from participant’s house, etc.) - Costs of mailing via secure courier - Inability to control testing environment (noise, etc.) - Inability to ensure participant follows study procedures (opening envelope prematurely, caregiver help, etc.)

However, telephone or other forms of remote NCT assessment may not be suitable for everyone. For example, patients with hearing impairment may need visual clues to understand testing instructions appropriately and patients with cognitive impairment may need closer supervision to ensure they have understood testing instructions. Moreover, some patients may prefer in-person administration in the presence of staff for supervision and support.

In comparison, testing through web-based or videoconferencing platforms allows real-time visual clues. However, there are several concerns limiting their real-life and widespread applicability, especially in the PD population. First, while nearly every individual possesses a telephone line, availability of the appropriate equipment and access to a quality high-speed internet connection required for operating web-based videoconferences may be difficult to obtain, especially in rural settings. Furthermore, some participants, especially those with cognitive impairment, may not possess the knowledge or have the capacity to learn how to operate the rapidly evolving technology. Telephone administration bypasses certain technological issues associated with web-based videoconferencing. Nevertheless, technical issues remain possible (e.g., poor sound quality or time lag) and some patients may also have difficulty using the telephone or the test materials without assistance. Overall, telephone administration of the MoCA appeared to be a more readily available and easily implementable option for NCT in in our population of PD patients with mild cognitive impairment.

Implementing remote NCT as part of a clinical trial involves several considerations. First, remote telephone testing generates additional costs due to mailing of the material using a secure courier. Videoconferencing also involves expenses as many videoconferencing platforms require licenses that can be costly. Moreover, careful orchestration is required to ensure the participant receives the testing material in due time and to coordinate the telephone visit accordingly. We have found a brief training session beforehand to be helpful to review the different steps with the participants and to instruct them on how to handle the testing material. Specific instructions should be given by the assessor to ensure the participant does not open the envelope containing the test materials ahead of time. Caregivers should be involved in the training and instructed not to assist participants during testing. In our study, only one participant was noted to receive help from his caregiver during one of the components of the NCT. The answer was appropriately discarded, and instructions reiterated. Involving the caregiver in every step of the process could prevent such issues.

Our exploratory analyses suggest that telephone administration of the MoCA test is reliable, although this should be reproduced in a larger sample. These results are aligned with previous studies evaluating web-based videoconference administration of the MoCA in movement disorders and PD [25, 26]. Abdholali et al. compared remote web-based videoconference-administration of the MoCA with baseline in-person testing in 17 patients with movement disorders, of which 7 had a diagnosis of PD [25]. They found good reliability for the total MoCA in the whole cohort but poor agreement between baseline and follow-up MoCA in the subset of PD patients. In addition to the small sample size, the longer delays between baseline and follow-up testing (7 months) may have explained the poorer agreement between remote and in-person MoCA in the PD cohort. In 2016, Stillerova et al. compared baseline in-person MoCA with videoconference administration conducted 1 week later in 11 patients with PD [26]. They found consistent results between evaluations and reported good participant and assessor satisfaction regarding the remote administration of the MoCA.

Alternative shortened versions of the MoCA, such as the MoCA BLIND, suitable for telephonic administration exist [16]. The MoCA BLIND, in which the visual elements of the original MoCA were removed, has good discriminative qualities for detecting mild cognitive impairment in patients following stroke [16, 17] but has not been assessed specifically in the PD population nor in repeat follow-up measures. However, to follow our original RCT protocol, we used the full MoCA, with different versions at different study timepoints, and mailed the visual portion of the test. Interestingly, the agreement between remote and in-person administration of the visual component of the MoCA was excellent.

Furthermore, apart from differences in the assessment of memory (RAVLT delayed recall) and some attention/working memory tasks (digit span backward, RAVLT immediate recall), remote administration of our neurocognitive assessment battery compared favorably with in-person NCT. For the RAVLT delayed recall, 3 of 4 participants in the remote NCT cohort substantially improved their score between baseline and 3 months. Due to the speed of progression through the NCT, it is highly unlikely that this was due to participants writing down the words. It is possible that improved recall was due to testing differences such as the familiar home environment or reduced stress compared to testing at the hospital, or to differences in the intervening testing between initial word encoding and RAVLT delayed recall. However, since some tests had to be omitted entirely, questionnaires were performed in lieu of those tests to maintain a similar delay for delayed recall. Although not extensively studied, the reported test-retest reliability of the RAVLT is variable, ranging from 0.12 to 0.86, and appears subject to minor practice effect [27, 28]. In 2012, a Brazilian study assessed the test-retest validity for each component on the RAVLT and found only a weak agreement (ICC 0.21) between the baseline delayed recall component of the test and the repeated measure at 35 (± 8.9) days [28].

Certain limitations must be considered when interpreting our results. Remote NCT was implemented as an alternative to in-person testing in light of COVID-19-related restrictions for in-person testing and was not specifically designed in the original COPE-PAP trial protocol. Our feasibility results may not be generalizable to older patients or those with more advanced PD. The current study’s primary objective was to describe the feasibility of substituting in-person with remote telephone NCT assessment in order to prevent data missingness and should not be perceived as a validation study. The sample size in the remote neurocognitive testing cohort is small, yielding large variability and imprecision of our estimates, and may have impaired our ability to detect statistically significant differences in the agreement between testing visits. Furthermore, the tests included in our modified neurocognitive assessment battery were not designed nor evaluated specifically for remote administration and larger validation studies are needed to ascertain their use in such context.

Finally, although informally deemed positive, patient acceptability of the procedure and preference was not formally evaluated as part of this study, but rather measured according to the participant’s willingness to undergo remote NCT. Moreover, obtaining participant’s feedback could have provided some insights as to why performance appeared to improve between baseline and 3 months. Future studies evaluating the feasibility of telephone neurocognitive assessment should take these aspects into account.

In conclusion, we report the feasibility of administering the full version of the MoCA test and a modified neurocognitive assessment battery over the phone to patients with PD and mild cognitive impairment. Prior to planning studies incorporating telephone-administered tasks, formal validation studies with a larger sample size should be conducted in order to specifically evaluate this remote testing method in this population.

## Supplementary Information

**Additional file 1: Supplemental table 1.** Agreement between baseline and 3-month MoCA and neurocognitive domains test components in control arm participants.

## Data Availability

The datasets used and/or analyzed during the current study are available from the corresponding author on reasonable request.
