# Supplementary material for: Feasibility of remote neurocognitive assessment: pandemic adaptations for a clinical trial, the Cognition and Obstructive Sleep Apnea in Parkinson’s Disease, Effect of Positive Airway Pressure Therapy (COPE-PAP) study
Source: Trials. 2021 Dec 11;22:910. doi: 10.1186/s13063-021-05879-1 (PMC8665856; doi:10.1186/s13063-021-05879-1)
Supplement: Supplementary file 1 — Additional file 1: Supplemental table 1. Agreement between baseline and 3-month MoCA and neurocognitive domains test components in control arm participants. [file 13063_2021_5879_MOESM1_ESM.docx]

**Supplemental table 1.** Agreement between baseline and 3-month MoCA and neurocognitive domains test components in control arm participants

| Component  (maximal possible score) | Remote NCT cohort  (n=4) | In-person NCT cohort  (n=25) |  |
| --- | --- | --- | --- |
|  | ICC  (95%CI) | ICC  (95%CI) | |
| Total MoCA score (30) | 0.60 (-0.43; 0.97) | 0.64 (0.34;0.82) ^*^ | |
| Visual component (8) | 0.84 (0.08; 0.99) ^*^ | 0.65 (0.36; 0.83) ^*^ | |
| Verbal component (22) | 0.57 (-0.46; 0.97) | 0.46 (0.10;0.72) ^*^ | |
| Attention and working memory | | | |
| Digit span – longest forward | 0.89 (0.41; 0.99) * | 0.66 (0.37;0.83) * | |
| Digit span – longest backward | -0.21 (-0.83; 0.72) | 0.60 (0.28; 0.80) * | |
| Digit span total (forward and backward) | 0.86 (0.29; 0.98) * | 0.84 (0.67; 0.93) * | |
| SDMT | 0.95 (0.66; 0.99) * | 0.66 (0.37; 0.83) * | |
| Total immediate recall (RAVLT) | 0.21 (-0.66; 0.87) | 0.54 (0.19; 0.76) * | |
| Executive functions |  |  | |
| Category switching accuracy (verbal fluency) | 0.78 (0.04;0.97) * | 0.58 (0.25; 0.79) * | |
| CWI inhibition | 0.80 (0.11; 0.98) * | 0.82 (0.62; 0.92) * | |
| CWI inhibition/switching | 0.77 (0.01; 0.97) * | 0.86 (0.70; 0.94) * | |
| Language |  |  | |
| Verbal fluency – letter | 0.48 (-0.44; 0.93) | 0.64 (0.34; 0.82) * | |
| Verbal fluency - category | 0.89 (0.38; 0.99) * | 0.82 (0.63; 0.91) * | |
| CWI – color naming | 0.89 (0.38; 0.99) * | 0.90 (0.79; 0.96) * | |
| CWI – word reading | 0.94 (0.61-0.99) * | 0.97 (0.94;0.98) * | |
| Memory |  |  | |
| Delayed recall (RAVLT) | 0.18 (-0.67; 0.86) | 0.73 (0.47; 0.87) * | |

*** Indicates statistically significant results (p <0.05). *Legend:* CI: confidence interval; CWI: Color-Word Inhibition; ICC: intraclass coefficient; MoCA: Montreal Cognitive Assessment, NCT: neurocognitive testing; RAVLT: Rey Auditory Verbal Learning Test; SDMT: Symbol Digit Modality Test
